# Perspectives of South African youth in the development of an implant for HIV prevention

**DOI:** 10.1002/jia2.25170

**Published:** 2018-08-27

**Authors:** Emily A Krogstad, Millicent Atujuna, Elizabeth T Montgomery, Alexandra Minnis, Sheily Ndwayana, Tsholofelo Malapane, Mary Kate Shapley‐Quinn, Kgahlisho Manenzhe, Linda‐Gail Bekker, Ariane van der Straten

**Affiliations:** ^1^ Desmond Tutu HIV Centre University of Cape Town Cape Town South Africa; ^2^ RTI International Women's Global Health Imperative San Francisco California USA; ^3^ RTI International Women's Global Health Imperative Los Angeles California USA; ^4^ Setshaba Research Centre Soshanguve South Africa; ^5^ Center for AIDS Prevention Studies University of California – San Francisco San Francisco California USA

**Keywords:** pre‐exposure prophylaxis, implant, adolescent, focus groups, product development, South Africa

## Abstract

**Introduction:**

Implants are a new dosage form in development for HIV pre‐exposure prophylaxis (PrEP) with potential for high adherence given that they are provider‐administered and are intended for long‐acting protection. Integrating end‐user preference into early stage product development may further overcome challenges with future product uptake and adherence. Hence, we sought to optimize the design of a PrEP implant in early‐stage development by gathering opinions about implant attributes from potential end‐users in South Africa.

**Methods:**

We conducted 14 focus group discussions (FGDs) with young women and men aged 18 to 24 in Cape Town and Soshanguve, South Africa, inviting participants into discussion as co‐designers. FGDs were homogenous by gender and previous implant experience. During FGDs, we showed prototype devices and followed a semi‐structured guide with questions on history of contraceptive implant use, preferences for physical characteristics of an implant, implant biodegradability, insertion process, participant‐driven ideas for implant design, and social adoption considerations. FGDs were facilitated in English, isiXhosa, Tswana, isiZulu, or Tsonga, audio‐recorded, transcribed into English, and qualitatively coded and analysed.

**Results:**

In this qualitative sample of 105 youth (68 women and 37 men), 58 participants were from Soshanguve and 47 from Cape Town, and 23% had previously used contraceptive implants. Participants expressed preferences for several implant design features; specifically, longer duration (≥6 months) was more important to most participants than the size or number of devices implanted. A majority preferred a flexible versus stiff implant to minimize palpability, thereby increasing discreetness and comfort. Nearly all participants favoured a biodegradable implant to avoid removal and thus reduce clinic visits. Concerns about the implant centred on its possible side effects and the “plastic” look of the prototype displayed for demonstration.

**Conclusions:**

This study offers preliminary insights into an implant for HIV prevention that provides long‐lasting protection may be well received among young South Africans. Additionally, flexibility, discreetness, and biodegradability may increase acceptability of the implant. Such end–user feedback is being incorporated into current implant designs in the hope of creating an effective long‐acting PrEP product that is likely to achieve high uptake and adherence in target populations.

## 1 Introduction

Pre‐exposure prophylaxis (PrEP) has the potential to protect millions of people from acquiring HIV, but recent clinical trials and demonstration projects have shown that poor adherence is a major barrier to actualizing this goal 1, 2, 3, 4, 5. Trials of PrEP administered in various formulations have identified several challenges to adherence, including user dislike of product attributes (e.g. leakiness of vaginal gels; size or taste of pills) 6, lack of partner support or ability to use a product discreetly if desired 7, 8, and misinformation and distrust of products and/or clinical research 9, 10. While acceptability studies may provide insight into future user uptake of PrEP products, many of these studies have been limited by social desirability bias, resulting in a positive view of products that may not reflect actual use patterns 11, 12, 13, 14. Consequently, there is a recognized need to better understand the end‐users’ context and true preferences for new PrEP products *before* they clinical trials are completed, and ideally, to include the voices of the end‐users earlier in product development 15, 16, 17, 18, 19, 20.

Subcutaneously inserted implants are a new PrEP dosage form in preclinical development with potential for greater adherence, given their long duration of protection and provider‐administered application. From a drug delivery standpoint, implants offer several advantages as a PrEP dosage form: stable drug release rates over a period of months to years, reversibility in the case of adverse events, and the avoidance of lingering sub‐therapeutic levels of drug in the blood. Several groups are currently developing implants for the delivery of antiretroviral drugs 21, 22, 23, 24, 25. The work presented here focuses on the thin film polymer device (TFPD) being developed by RTI International and colleagues, a biodegradable reservoir implant 26, 27. Since the TFPD PrEP implant is still in preclinical development and has several attributes that can be modified (e.g. size, appearance, stiffness, biodegradability), we aimed to elicit feedback from potential female and male end‐users in South Africa on their perspectives and preferences for these modifiable attributes. Specifically, we focused on South African youth ages 18 to 24 as potential end‐users of a PrEP implant as they are at increased risk for acquiring HIV 28, 29.

## 2 Methods

### 2.1 Setting

Data collection occurred at two sites in South Africa between December 2016 and June 2017: Desmond Tutu HIV Research Centre/Foundation (DTHC/F) in Cape Town and Setshaba Research Centre (SRC) in Soshanguve. We selected these two geographically distinct locations to explore variation in end‐user preferences based on different local cultural and community dynamics. In both locations, participants were from densely populated townships that were established during apartheid. Study participants in both Cape Town and Soshanguve described the community environments as characterized by high rates of HIV, unemployment, violence, and poverty, consistent with nationally reported statistics 30, 31, 32, 33, 34. District‐level HIV prevalence among pregnant women at public antenatal clinics in 2015 was 21.6% for Cape Town and 25.3% for Soshanguve 35. Cape Town participants were from areas predominantly inhabited by Xhosa people originating from the Western or Eastern Cape 36. Soshanguve is approximately 30 km north of Pretoria and has high multilingual and ethnic diversity, with Setswana, Sesotho, and Sepedi as primary languages 37.

### 2.2 Study design

We conducted 14 focus group discussions (FGDs), designed to be highly interactive and create an environment where participants were encouraged to provide feedback directly into product development as “co‐designers”. To accentuate this co‐designer role, we began FGDs by offering participants laboratory coats to wear during the discussion, a technique that was pretested before implementation. We explained that the implant was still in the early stages of development, and that the laboratory coats were intended to symbolize the importance of their input as fellow scientists and as representatives of their community into product design.

**Figure 1:**
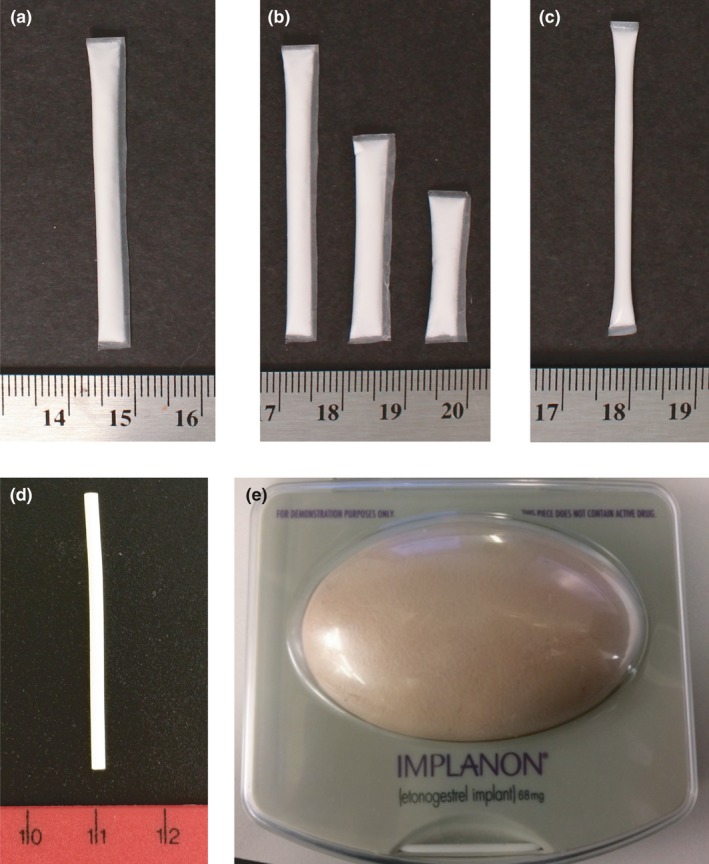
Physical prototypes shown during FGDs. **(a)** TFPD implant in development, a reservoir device with a biodegradable polycaprolactone (PCL) membrane fabricated using a solvent casting technique. **(b)** Multiple sizes of TFPD implants in development. **(c)** Another type of TFPD implant in development, made using an extruded tube fabrication technique. The extruded tube prototype was only shown in 7/8 FGDs in Soshanguve; not available for Cape Town FGDs. **(d)** Implanon NXT^®^ contraceptive implant. **(e)** Model of Implanon NXT^®^ palpability under mock skin. Note that all TFPD prototypes shown during FGDs contained cellulose powder as placebo in place of active drug. Actual TFPD prototypes include a liquid excipient, resulting in a drug slurry paste within the implant instead of a white powder.

The structured FGD guide addressed five themes: perceptions of or actual experience with contraceptive implants, physical attributes of PrEP implants, implant insertion, a “design‐your‐own” implant activity, and social adoption considerations. Themes were explored through group discussion and follow‐up probes. Participants were given examples to see and touch during discussion, including multiple prototypes of TFPD implants currently in development, Implanon NXT^**®**^ contraceptive implants, and a model of how Implanon NXT^**®**^ feels under mock skin (Figure 1). Pictures of implant insertion and TFPD biodegradation processes were also shown (Figures S1 and S2). We obtained feedback on participants’ preferences for various attributes through five voting exercises in which participants placed a sticker along a continuum with two extremes (e.g. flexible vs. stiff implant) and discussed their choices.

### 2.3 Participant selection

We used a recruitment approach maximizing variation by categorizing FGDs based on gender, contraceptive implant experience, PrEP injection experience, and geographic location (Table 1). Eligibility criteria included being 18 to 24 years old, sexually active (had sex at ≥3 times in the past three months), and HIV negative based on self‐report. Participants were recruited from the community through word‐of‐mouth and direct outreach activities. This study builds on the iPrevent and TRIO studies 38, 39. Some Cape Town participants were recruited through re‐contacting former iPrevent study participants (*N *=* *3) 38. In Soshanguve, three FGDs were held with PrEP injectable‐experienced women who were former TRIO study participants 39, providing unique insights into PrEP dosage form comparisons.

**Table 1:** FGD categories by study site (*n *=* *105 total participants)

Participant group	Study site	
	Cape Town, SA	Soshanguve, SA
Women (age 18 to 24) Implant‐naive (and PrEP injectable‐naive^a^)	*N* = 2 FGDs (*n* = 8; *n* = 10)	*N* = 2 FGDs (*n* = 8; *n* = 5)
Women (age 18 to 24) Implant‐naïve (and PrEP injectable‐experienced^a^)	*n*/a	*N* = 2 FGDs (*n* = 7; *n* = 6)
Women (age 18 to 24) Implant‐experienced^b^	*N* = 2 FGDs (*n* = 8; *n* = 4)	*N* = 2 FGDs (*n* = 5; *n* = 7)
Men (age 18 to 24) Implant‐naive	*N* = 2 FGDs (*n* = 7; *n* = 10)	*N* = 2 FGDs (*n* = 12; *n* = 8)

*N*, number of FGDs; *n*, number of participants per FGD; PrEP, pre‐exposure prophylaxis; FGDs, focus group discussion.

a PrEP‐injectable experienced versus PrEP‐injectable naïve was only a stratifying FGD group criteria in Soshanguve, not in Cape Town. PrEP injectable‐experienced women had previously received two shots (×2 mL) of saline (placebo), one in each gluteal muscle, as part of the TRIO study 39.

b Implant‐experienced women had previously used either the Implanon NXT^**®**^ (publically available in South Africa) or the Jadelle contraceptive implant (available as part of clinical trials in South Africa).

### 2.4 Data collection and analysis

Prior to finalization of the FGD guide, three mock FGDs were held at each site to further refine questions and procedures. Participants first completed informed consent followed by an interviewer‐administered demographics questionnaire. FGDs in Cape Town were co‐facilitated by a social scientist (SN) and a bioengineer (EK) in English and/or isiXhosa and FGDs in Soshanguve were facilitated by a social scientist (TM) in English, Tswana, Tsonga and/or isiZulu. FGDs were audio‐recorded and lasted approximately two hours each. Facilitators wrote debriefing reports documenting key findings under each interview topic for every FGD.

**Figure 2:**
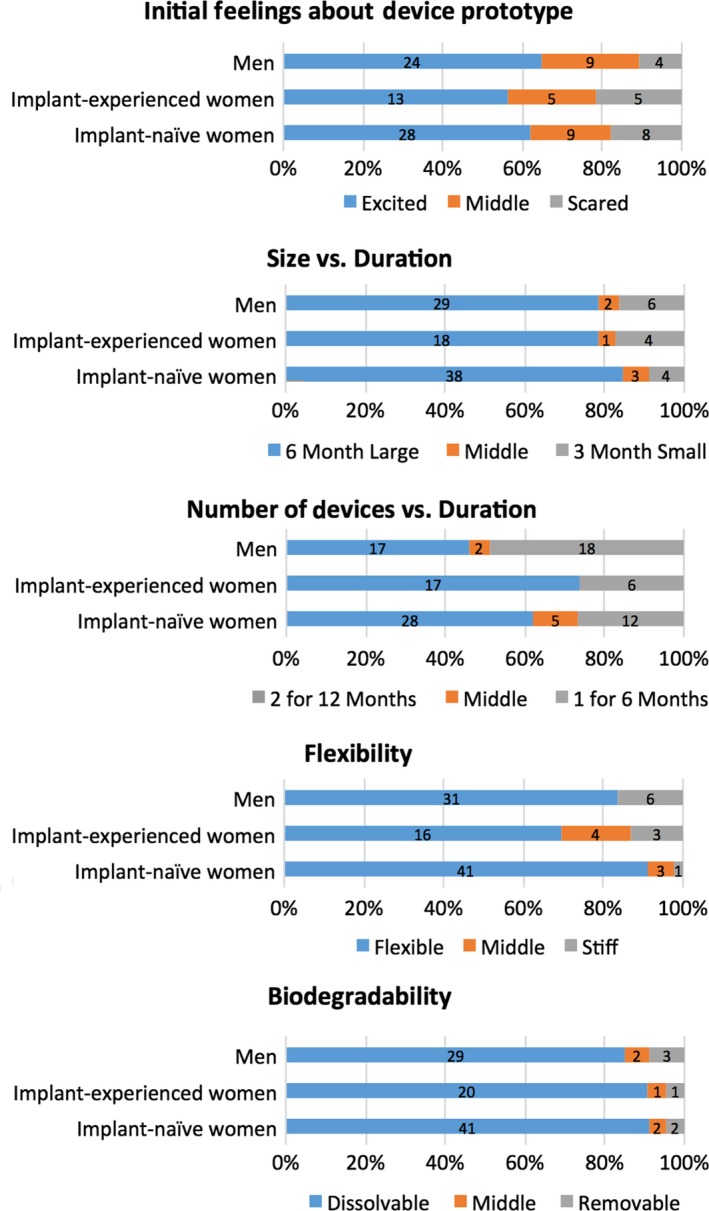
PrEP implant attribute preferences from FGD voting statement activity, stratified by FGD group category (*N *= 105 total participants). Reflects individual voting responses from all participants when asked to indicate their preferences for product attributes by placing a sticker along a continuum with two extremes representing differing attribute levels. Although participants were allowed to place stickers anywhere along a continuum for the voting statement activity, we used a quantitative approach to analyze results by assigning votes to one of three categories: (1) preferred attribute on the left end of continuum, (2) preferred attribute on the right end of continuum, or (3) “middle” if participant placed the sticker in between the two extremes, indicating either that the participant preferred a compromise or middle level of given attributes, was undecided, or preferred none of the options given. Numbers on bars indicate *n* = number of participants who gave that response.

FGDs were transcribed and translated into English, and transcripts were reviewed for accuracy. Transcripts were coded using Dedoose software 40 by two researchers. The coding process involved defining codes based on key themes, regularly discussing code applications to ensure consistency among the coding team, and iteratively refining code definitions. Qualitative analysis was done through content analysis of debriefing reports of FGDs using matrices to facilitate comparison of distilled findings of themes across FGD subgroups, and through summary memos on code reports of key codes. Voting activity results were analysed quantitatively as described in Figure 2.

### 2.5 Ethical approval

This study was approved by the Institutional Review Boards at all participating institutions. All participants provided written informed consent and were reimbursed ZAR 100 (approximately 7 USD) for their time and transportation.

## 3 Results

This study included 105 participants across 14 FGDs, with 6 FGDs (*n *=* *47 participants) in Cape Town and 8 FGDs (*n *=* *58 participants) in Soshanguve. Demographic characteristics across both sites were relatively similar, with the exception of ethnic group (Table 2). Our sample was comprised of 65% females and 35% males, with 23 implant‐experienced females, 44 implant‐naïve females and 37 implant‐naïve males. The most salient attributes of implants that emerged from the FGDs are thematically presented below, with illustrative quotes in text or Table 3.

**Table 2:** Background characteristics of FGD participants, stratified by site

	Soshanguve	Cape Town	Total
Basic demographics			
Total participants – *n*	58	47	105
Age (years) – mean	21.1	21.1	21.1
FGD group – *n* (%)			
Male	20 (35)	17 (36)	37 (35)
Female – implant‐naive^a^	26 (45)	18 (38)	44 (42)
Female—implant‐experienced	12 (21)	12 (26)	24 (23)
Ethnic group – *n* (%)			
Zulu	17 (29)	1 (2)	18 (17)
Xhosa	0 (0)	42 (89)	42 (40)
Sotho	21 (36)	1 (2)	22 (21)
Tswana, Tsonga or Pedi	16 (28)	0 (0)	14 (13)
Currently receive own source of income – *n* (%)	14 (24)	16 (34)	30 (29)
Highest level of education – *n* (%)			
Primary school, complete	1 (2)	0 (0)	1 (1)
Secondary school, not complete	18 (31)	13 (28)	31 (30)
Secondary school, complete	24 (41)	13 (28)	37 (35)
Attended college or university, not complete	14 (24)	16 (34)	30 (29)
Attended college or university, complete	1 (2)	5 (11)	6 (6)
Has ever had children – *n* (%)	21 (36)	15 (32)	36 (34)
Current relationship status – *n* (%)			
Married	1 (2)	1 (2)	2 (2)
In a partnership (not married)	40 (69)	40 (85)	80 (76)
Single	17 (29)	6 (13)	23 (22)
Co‐habiting with main partner	8 (14)	8 (14)	16 (15)
Sexual risk factors			
Number of sexual partners – mean (range)			
In lifetime	6 (1 to 18)	4.6 (1 to 11)	5.4 (1 to 18)
In past 30 days	1.1 (0 to 4)	1.4 (0 to 5)	1.2 (0 to 5)
Currently has main sexual partner – *n* (%)	44 (76)	44 (94)	88 (84)
Currently has casual sexual partner(s) – *n* (%)	22 (38)	15 (32)	37 (35)
Thinks partner has other partners – *n* (%)			
Yes, I know	2 (5)	5 (11)	7 (8)
Yes, I suspect	9 (21)	13 (30)	22 (25)
Do not know	17 (40)	13 (30)	30 (34)
No	16 (36)	13 (30)	29 (33)
Condom usage at last sex act – *n* (%)	37 (69)	25 (60)	62 (65)
Previous product experience			
Ever used for HIV or pregnancy prevention – *n* (%)^b^			
Pills	11 (19)	16 (34)	27 (26)
Injectable	28 (48)	23 (49)	51 (49)
Implants	14 (24)	14 (30)	28 (27)
Gel/spermicide	1 (2)	2 (4)	3 (3)
Ever had contraceptive implant removed – *n* (%)^c^	2 (17)	4 (33)	6 (25)
Months current contraceptive implant in – mean (range)^c^	22.9 (15 to 30)	14.4 (1 to 36)	19.1 (1 to 36)

FGD, focus group discussion.

a One participant in an implant‐naive female FGD in Cape Town revealed partway through the FGD that she had previously used a contraceptive implant.

b More than one response allowed; includes dosage forms their partner(s) have used

c Only contraceptive implant‐experienced participants responded to these questions; percentages reflective of total number of contraceptive implant‐experienced participants only.

**Table 3:** Participant quotes on key themes of PrEP implant prototype attributes

Theme	Quote^a^
Implant duration	“I chose 3 months. I want to see first how it is when it is in my body. So that I don't take 6 months which is long when I don't know how it's going to be in my body […] I have to see first and then if it does me bad or well, that's when I would take this one [longer duration].” *Lulu, Implant‐experienced female, Cape Town*
Concerns about implants	“This plastic [thin film portion of PrEP implant prototype] […] I think it's the one thing that is going to confuse them [community], just like it confused me. I think many are going to start with that issue that ‘Exactly how does it dissolves in the underneath the skin?’ Like even if you can explain it, they would still want to experience it first. ‘Let me see, did it really dissolve, but are you sure that it has dissolved?’ Such things.” *Nomfundo, Implant‐experienced female, Soshanguve*
	“In 2016 there was the habit of junkies smoking this thing [contraceptive implant]. You would be stabbed on where the implant is located and they would take it out […] Yes I was once grabbed by a person and, ‘Why are you holding me?’ And he said, he was saying, ‘Did you not have those things [contraceptive implant] inserted?’ And I said, ‘No I'm still young, I'm scared of them, why?’ He then said, ‘By the way we use these things and we smoke them.’” Lira, Implant‐experienced female, Cape Town
	“That medicinal drug that is within that [contraceptive] implant is the one […] that gives it more of a kick, that makes their addicts go sky high.” *Jeffrey, Male, Cape Town*
Implant flexibility	“The reason why I feel excited is because it will be invisible when I have inserted it [PrEP implant]. Nobody is going to see that it is there, unlike the one for pregnancy prevention” *Hlumelo, Implant‐naïve female, Soshanguve*
	“I would go with the flexible one because like for instance, have you ever had stitches in? Like we are human beings – for instance like when that one if I can feel it, I'll forever be busy touching it, pushing it around. So if I can't feel it, I won't, I won't even know that it's there.” *Mpho, Male, Soshanguve*
	“I would say I'm not sure whether I like it stiff. If I choose stiff my worry is to be robbed because it can be felt from outside that there is an implant just like now. They [robbers] feel and rob contraceptive implants. They know that it has drugs. If I choose the flexible one my worry is that I won't be able to feel its whereabouts maybe it has moved to the neck maybe or where.” Lunitha, Implant‐experienced female, Cape Town
Implant biodegradability	“Many people are complaining about the [contraceptive] implant. I even saw it on Checkpoint [reality TV program]. They are talking that they are having periods for a long time and when they go to the clinic to ask them remove the implant, the nurses tell them that ‘we don't – we can't remove it because we are not trained to remove it, we are only trained to insert it.” *Asive, Implant‐naive female, Soshanguve*
	“Are the nurses going to be trained about it on how to place it [PrEP implant] and how to remove it? Because on the pregnancy [contraceptive] implant nurses didn't know how to remove the implant when people wanted it to be removed. So that becomes a problem to us when you go to them and say, ‘Okay now I want to it to be removed’. They don't know how to remove it. So we need them to be extremely trained.” *Ugly Rapper, Male, Cape Town*
	“I still feel that the community is still going to criticize that device; they are some people who are going to say negative things about it that ‘no, this thing is not working’ or maybe ‘you are going to get sick in future’ or whatever […] They will talk, they will say negative things about it. They are going to say that ‘it [biodegradability] is not good, how they can insert it and not remove it, it stays inside?’ They are going to ask ‘what's going to happen to it once inside?’” *Asive, Implant‐naive female, Soshanguve*

a Names used in this table are pseudonyms, not actual participant names.

### 3.1 Implant duration versus dimensions

When asked to compare trade‐offs between implant duration, dimensions, number of rods inserted at a time and dosage form, longer duration of protection emerged as the strongest driver of product preference. Participants wanted to reduce inefficient and time‐consuming clinic visits and avoid needing to remember to take a product frequently:

First of all I'm tired of pills. Secondly, I do not want to go every 6 months. I want it to last for longer. […] That is why I chose the 12 months one because it's actually going to stay in my body and do its job slowly but surely. (Ugly Rapper, Male, Cape Town)

A majority of participants voted for a longer duration PrEP implant when presented with choices between a) three‐month small implant and six‐month large implant, and b) six‐month single rod versus two rods that would last 12 months (Figure 2). Additionally, when presented a choice between a three‐month PrEP injection (four times/years) versus a 12‐month PrEP implant, most participants preferred an implant due to its longer duration.

When probed further on desired dosing frequency, participants suggested an ideal duration of one to three years, but felt that an implant lasting three to six months would still be acceptable. A minority of participants preferred a device with a shorter duration, as it would have the benefit of encouraging more frequent testing for HIV/STIs and access to clinic services. Notably, others specifically suggested the option of shorter duration devices (three to six months) because they wanted “*to see first how it is when it is in my body*” before committing to a longer duration device.

### 3.2 Concerns about PrEP implants

Participants were encouraged to ask questions throughout the FGDs, and the most common concerns were related to expected side effects and efficacy. Some participants were concerned that having two rods (vs. one rod) could result in more side effects or drug overdose. Others wondered how daily activities like weight lifting, dancing, or chores would influence drug release and efficacy. Some shared concerns around the time required for drug to travel from the location of insertion to target tissues for HIV prevention (e.g. arm to vagina), as well as where the physical components of the device go in the body as it biodegrades.

When discussing perceptions of contraceptive implants, participants shared stories circulating in the community. In Soshanguve, the most common stories related to side effects experienced by women using the contraceptive implant (e.g. menstrual bleeding changes, weight changes, headaches, sore arm), lack of efficacy and implant migration within the body. Similar stories were also discussed in Cape Town. Further, in five of the six FGDs in Cape Town, the dominant story was that implants were being robbed and physically cut out from women's arms by drug users who smoke them to get high.

Considerable concern was raised about the appearance of the thin film membrane of the device that for many looked like “*plastic*”. Some thought the prototype device appeared like street drugs in a plastic bag, exacerbating the worries related to implant robbery in Cape Town. Others questioned the safety of “*plastic*” for insertion in the body and how “*plastic*” would dissolve. One implant‐experienced woman from Soshanguve suggested giving the thin film “*plastic*” portion of the device a specific name to reduce concern, “*because once you think of plastic, you think of death*”.

Anticipated pain during implant insertion was initially concerning among implant‐naïve women and men, but not among implant‐experienced women. However, after hearing an explanation of the numbing medication used during insertion, most implant‐naïve participants were no longer concerned about pain. One FGD in Soshanguve had five participants who had all previously used a contraceptive implant, as well as placebo PrEP injections. When asked to compare actual pain experienced between a PrEP injection and contraceptive implant, all five agreed that the implant was less painful and indicated preference for an implant over an injection for future PrEP.

### 3.3 Flexible versus stiff implants

After having the opportunity to feel an Implanon NXT^**®**^ under imitation skin in a model arm, most participants preferred a softer, more flexible implant that would be less likely to be palpable by others, making it more discreet and more “*invisible*” compared to a stiff device. This invisibility was characterized by three aspects: (1) not visible in appearance from the outside, (2) not able to be felt under the skin (palpated) by others, and (3) forgetable/not distracting to themselves. Others preferred a soft device because they perceived it to be more “*comfortable*” both mentally (forget its presence; not worry about others feeling it) and physically (perceived as less irritating inside body). Although many participants would disclose PrEP implant use to friends, family and/or partners so that they could also be protected, most men and women preferred an implant that could be used discreetly if desired, as described here:

I chose the soft one because I don't want to feel it. […] I don't want to be reminded each and every time that it's there, you see. And also I want it to be my own private thing. And only the person I have told that I have it should know, not for everyone to see. (Babalwa, Implant‐naïve female, Soshanguve)

A minority preferred a stiffer device so they could self‐monitor device movement, influenced by a community misconception that the implant can migrate in the body to the lungs, neck, or other distant location. Participants in Soshanguve voiced these concerns more strongly than those in Cape Town, and thus a higher proportion from Soshanguve expressed a preference for a stiffer implant. In contrast, Cape Town participants discussed a preference for a flexible implant because it was perceived to be less visible and/or palpable to robbers.

### 3.4 Dissolving versus non‐dissolving implants

TFPD implants are fabricated with a biodegradable polycaprolactone membrane and may remain intact through the therapeutic window before biodegrading in the body 26. After explaining the concept of biodegradability using several analogies, participants expressed a clear preference for a biodegradable implant that does not require removal. The rationale for preferring biodegradability echoed that for preferring a longer duration product: participants wanted to reduce the number of visits and time at the clinic, and avoid the removal process that was perceived as painful and often described as “*being cut”*.

I think the dissolving one is perfect. Because I won't have to be cut again to remove it. As I said before I don't get along with pain. (O.S., Male, Cape Town)

Concerns were also raised at both sites regarding an existing lack of trained providers to remove contraceptive implants, and, therefore, a lack of expertise to offer removal services for a future PrEP implant. While most participants, after explanation, seemed to understand the concept of biodegradability and were excited by it, some participants raised concerns that the community may have a hard time understanding or accepting a device that dissolves in the body. Participants emphasized that education and counselling alongside biodegradable PrEP implant rollout would be necessary.

### 3.5 Participant‐driven recommendations for design of PrEP implants

Overall, participants responded enthusiastically to the opportunity to be involved in design of new technology for HIV prevention as part of these FGDs, with one participant summarizing: “*We should take initiative of our own health, and we should take forefront to come up with solutions for our own health*.” Many participants were visibly excited by the opportunity to wear lab coats during the FGDs (none were observed to react negatively) and several emphasized that their feedback be shared with researchers developing the implant.

**Figure 3:**
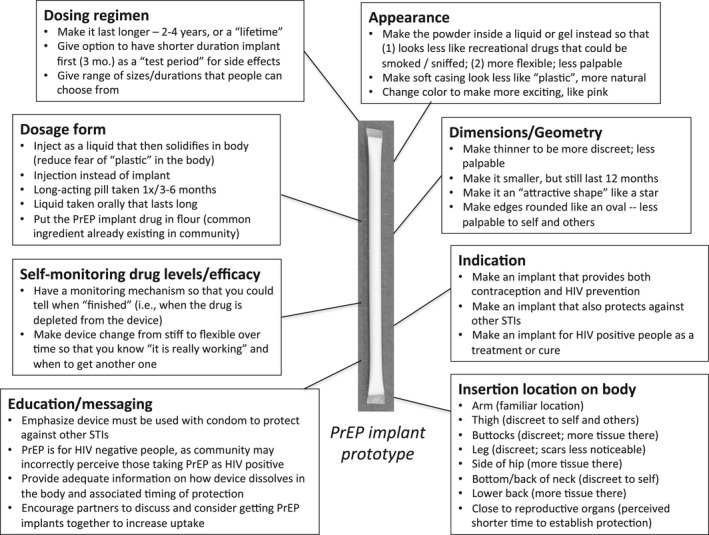
Participant‐driven “co‐designer” recommendations for PrEP implant design. Summary of participant suggestions for how to change the device when asked, “If you were the scientist, how would you design the PrEP implant?”.

When asked to design their own implant as co‐designers after interacting with prototypes, participants gave several suggestions consistent with the themes presented above (Figure 3). This included extending the duration of protection to over one year, making the device thinner and more flexible to be more hidden, and reducing the “plastic”‐like appearance. In Cape Town in particular, several participants suggested changing the design to be more discreet to robbers, such as changing the physical appearance of the white powder drug because “*they are going to think it's cocaine*” (see note in Figure 1), or placing it in another location besides the arm, such as the thigh, buttocks, or side. Additionally, several participants emphasized a modular approach, offering choice between different types of implants. They recommended that people be able to choose their preferred size and duration (e.g. large vs. small; three months vs. six months) as well as their preferred indication (e.g. HIV prevention, dual HIV prevention and contraception, or prevention of other STIs).

## 4 Discussion

In this qualitative study conducted with youth aged 18 to 24 at two South African sites, we elicited end‐users’ perspectives to better understand what modifiable attributes are most important in an HIV PrEP implant that is still in pre‐clinical development. Overall, most participants preferred an implant that is long‐acting (>3 to 6 months at minimum, but >1 year ideally), can be used discreetly (e.g. invisible by appearance and by palpation), and minimizes interaction with the health care system (e.g. biodegrades at end of duration to avoid removal). This consensus in preference across multiple user groups is especially noteworthy given that we selected a recruitment approach to maximize variation. These results suggest that a long‐acting, flexible, biodegradable implant may serve as an acceptable dosage form for PrEP with potential to maximize young users’ uptake and adherence.

Surprisingly, the physical appearance of the implant emerged as an attribute that mattered to many participants, even though an implant is inserted subcutaneously and is not typically externally visible. The negative connotation of the plastic‐like appearance of the implant prototype came up strongly and consistently across both sites. In response to participant preferences and suggestions on changing the “plastic” appearance in this study and other technical considerations such as manufacturability, TFPD developers are currently changing the design of the TFPD to be more rod‐shaped and streamlined to reduce the “plastic” overhang present in earlier prototypes. Identifying such modifiable attributes early on in product development and re‐centring design around user preferences is critical to product developers to consider and to maximize future user acceptability 15, 16, 17.

Though helpful to understand what an ideal PrEP implant would look like from the user perspective, end‐user preferences must also be grounded in practical design constraints from an engineering perspective. Hence, through voting activities, we assessed the relative weight that participants placed on a range of product attributes to prioritize key features in design. When presented with trade‐offs, long‐lasting duration emerged as most salient over other attributes such as rod dimensions, number, or dosage form (injection vs. implant). Other studies have similarly reported longer duration being a strong driver in HIV prevention and/or contraception dosage form preference 39, 41, 42, 43, including follow‐up studies with women who have previously used vaginal rings 44, oral PrEP, or vaginal gels 45. Since duration weighed so heavily relative to other attributes for FGD participants, developers are prioritizing achieving longer duration for the next iterations of TFPD implants, which may require insertion of two rods instead of one rod.

An understanding of the context of the user experience helps to provide an explanation for the product preferences that emerged in this research. Many participants described barriers associated with the South African health care system, including long queues, transportation costs, privacy concerns and the lack of trained providers to remove implants. This is consistent with previous reports on barriers in the South African health care system among adolescents 46, 47 and inadequate training on contraceptive implant removal 48, 49. Such barriers motivated overall participants’ preference for a long‐acting, biodegradable implant that would reduce clinic interactions.

We found that preferences for various implant attributes were remarkably similar across user groups defined by gender, product experience and geographic location within South Africa. Despite different community stories about contraceptive implants (implant robbery stories only emerged in Cape Town, whereas concerns about implant migration were more dominant in Soshanguve), overall preferences for device attributes were consistent across location. However, myths and misconceptions about products have an important influence on uptake and adherence 8, 50, and engineering can only go so far in addressing end‐user concerns, particularly those that emerge after key decisions in design have been finalized. As several participants themselves suggested, this draws focus to the importance of identifying and addressing community‐specific concerns and misunderstandings throughout all stages of product development. Understanding the community perceptions of new technology highlights the value in conducting multisite research, even for exploratory qualitative studies, to understand the local context for product introduction and to be able to proactively respond to misinformation.

This study was innovative in both the early timing in the product development lifecycle during which end‐users were involved (preclinical) and the FGD activities used to engage participants as “co‐designers”. To our knowledge, this was the first study where potential end‐users from South Africa provided feedback on prototypes of implants for HIV prevention, and demonstrates the importance of conducting such research with users from the context of intended product use. However, it is important to recognize that what participants say about hypothetical product use in a focus group setting may not reflect actual real‐world use. Focus groups may also be limited by one person dominating the discussion and some participants being reluctant to express a divergent opinion during voting activities. In this study, we attempted to overcome limitations of hypothetical end‐users by grouping FGD participants based on contraceptive implant experience and gender, and found that overall, preferences were relatively similar across groups. Further, we included prototype interaction activities, detailed explanations, pictures, and analogies to give participants adequate baseline knowledge and exposure to the PrEP implants in development. Future end‐user studies will be necessary as this and other HIV PrEP implants advance along the developmental pipeline to confirm these findings, as well as to explore similarities and differences across other sites and populations.

## 5 Conclusions

We present early evidence that a diverse South African youth population may prefer a PrEP implant that is long‐lasting, flexible, biodegradable and not visible. The timing of this study during the preclinical stage of development and “co‐designer” methodology provides a framework for others interested in seeking methods to engage end‐users in product development for applications like HIV prevention, contraception and multipurpose prevention technologies.

## Competing interests

The authors report no conflicts of interest.

## Authors’ contributions

E.K., M.A., E.M., A.M, L.‐G.B. and A.v.d.S. designed the research study. E.K., S.N. and T.M. conducted the research. E.K., T.M., K.M., S.N., and M.K.S.‐Q. coordinated and managed data collection during the study. E.K. and M.K.S.‐Q. analysed the data. E.K., M.A., E.M., A.M. and A.v.d.S. wrote the manuscript. All authors have read and approved the final manuscript.

## Supporting information


**Figure S1.** PrEP implant biodegradability pictorial tool and analogies.
**Figure S2.** PrEP implant insertion pictorial tool.
