# Supplementary material for: Perspectives of South African youth in the development of an implant for HIV prevention
Source: J Int AIDS Soc. 2018 Aug 27;21(8):e25170. doi: 10.1002/jia2.25170 (PMC6111144; doi:10.1002/jia2.25170)
Supplement: Supplementary file 1 — Figure S1. PrEP implant biodegradability pictorial tool and analogies. Figure S2. PrEP implant insertion pictorial tool. [file JIA2-21-e25170-s001.docx]

**Supporting Information**

Manuscript: “Perspectives of South African youth in the development of an implant for HIV prevention.”

E.A. Krogstad, *et al*


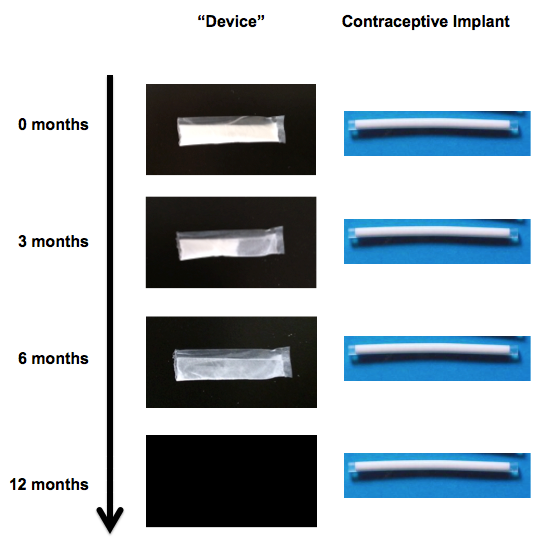


**Supplementary Figure 1. PrEP implant biodegradability pictorial tool and analogies.** Pictorial tool used during FGDs to describe TFPD PrEP implant biodegradation process. Three analogies were used to describe the process of biodegradation to FGD participants: (1) a sweet dissolving in the mouth (participants were given an Endearmint**®** or similar hard candy to eat during the explanation of this analogy), (2) a seed growing in soil, and (3) an oral pill capsule being digested by the body. Analogy (1) was described in all FGDs, and analogies (2) and (3) were used in addition for some FGDs depending on time and perceived participant level of understanding.


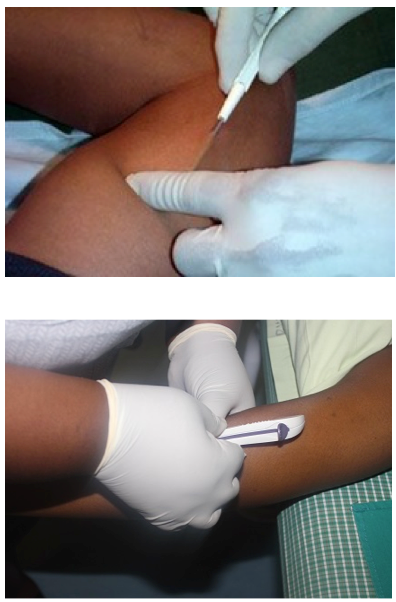


**Supplementary Figure 2. PrEP implant insertion pictorial tool.** Pictorial tool used during FGDs to show implant insertion process.
